# Waking up to the alarm: sleep, clocks, and making memory (s)tick

**DOI:** 10.3389/fnsys.2015.00065

**Published:** 2015-04-22

**Authors:** Jason R. Gerstner, Sara J. Aton, H. Craig Heller

**Affiliations:** ^1^College of Medical Sciences, Washington State UniversitySpokane, WA, USA; ^2^Molecular, Cellular, and Developmental Biology, University of MichiganAnn Arbor, MI, USA; ^3^Department of Biology, Stanford UniversityStanford, CA, USA

**Keywords:** sleep, circadian rhythms, memory, plasticity, learning, synapse

In the immortal words of Woody Allen, “time is nature's way of keeping everything from happening at once.” There is a time to work, a time to learn, and a time to rest. But in an increasingly 24-h society, the question of why *we must rest* comes up. Sleep and circadian rhythms influence brain plasticity-related processes, including neural excitability, synaptic efficacy, and cognitive abilities, such as learning and memory. How (and from an evolutionary perspective, why) sleep and the circadian clock have such influences over the brain is one of the great unsolved mysteries of biology. Clues regarding these interactions have been observed throughout the animal kingdom, and suggest basic mechanisms by which sleep and the circadian system that govern these processes are conserved phylogenetically. This Research Topic highlights current research and views on sleep and chronobiological features of plasticity and memory in multiple species, models, and systems. The authors present original research using invertebrate and vertebrate species, including moths, flies, rodents, and humans, giving the reader a broad understanding of available models and systems. Review articles discuss functional consequences of sleep and circadian disruption on cognitive processes, and survey current ideas within this burgeoning field of neuroscience. This Research Topic will hopefully stimulate more research inquiry and open the door for improving our understanding of relationships between sleep, chronobiology, and cognitive function.

Michel and Lyons (2014) underscore the importance of using invertebrate model systems for examining relationships between sleep, clocks, and memory. They show that vertebrate and invertebrate species, while separated by hundreds of millions of years of evolution, share common molecular, and cellular mechanisms that shape complex behavior and plasticity processes. Conservation of these basic mechanisms may have emerged out of ancient adaptive processes first directed by circadian processes to better equip species for survival, leading to testable hypotheses in multiple organisms (Gerstner, 2012). Available genetic and molecular tools, combined with strong phenotypes and cost-effectiveness, make invertebrate species powerful animal models for investigating mechanisms underlying complex behaviors, such as chronobiological aspects of memory formation.

Two original research articles harnessed the power of invertebrate model systems to reveal time-of-day effects on memory formation. Gage and Nighorn (2014) provide evidence for nitric oxide (NO) in the diurnal regulation of olfactory memory in the hawkmoth, *Manduca sexta*. Using the established proboscis extension reflex paradigm, a type of appetitive classical conditioning in *M. sexta*, the authors show NO-signaling has strong time-of-day effects on short- and intermediate-term memory formation. Fropf et al. (2014) use the olfactory avoidance conditioning paradigm in the fruit fly, *Drosophila melanogaster*, to characterize time-of-day effects on long-term memory formation. The authors show that these time-of-day dependent differences in memory performance are associated with changes in specific activation states of the protein dCreb2, a transcription factor implicated in sleep, circadian rhythms, and memory formation. These studies feature invertebrate models to characterize molecular signaling cascades which contribute to time-of-day dependent changes in memory formation, and lay groundwork for future studies to test whether similar pathways are conserved phylogenetically.

Two original research articles employ studies in a mouse model system to assess how sleep and the clock regulate neurophysiology. Ognjanovski et al. (2014) present data on sleep- and wake-associated changes in CA1 hippocampal network activity during memory consolidation. While previous studies have described the necessity of sleep following single-trial contextual fear conditioning, the effects of conditioning and subsequent sleep on network activity have not been well understood. Here, Ognjanovski et al. (2014) show that consolidation of contextual fear memory is accompanied by heightened neuronal firing in the hippocampus. The authors observed that hippocampal network stability, as measured by functional connectivity analysis of neuronal spike trains, was greater after conditioning, specifically, during post-conditioning slow wave sleep, suggesting sleep may play a role in stabilizing patterns of neuronal communication following new learning. Gerstner et al. (2014) provide the first evidence that seizure threshold in mice is regulated by circadian clock mechanisms. Using a step-wise electroshock paradigm, the authors found that seizure thresholds peak in the early dark phase (the beginning of the active period), and that the core-clock gene BMAL1 is responsible for this effect, suggesting molecular clock mechanisms are able to regulate baseline neural excitability. Together, these data suggest that sleep and clock molecular factors are able to regulate neuronal network activity in mammalian brain, and provide novel models in rodents from which to explore mechanisms relating sleep and the clock in activity-dependent plasticity-related processes.

Two review articles elaborate on the use of rodent models for studying interactions between the clock, sleep, and brain function. Iyer et al. (2014) review comparisons between circadian plasticity mechanisms in the hippocampus and the master circadian pacemaker, the suprachiasmatic nucleus. The authors suggest circadian neuronal plasticity is gated by endogenous clock mechanisms, forming the basis for ~24 h *iterative metaplasticity*, a term describing daily temporal confines to synaptic plasticity. Colavito et al. (2013) review rodent models in the study of sleep dependent memory processing. Here, the authors provide an extensive history on sleep deprivation methods to help facilitate interested researchers for developing customized laboratory protocols, and their application to preclinical testing. These articles highlight the use of rodent models to study interactions of sleep and circadian systems with brain plasticity and memory formation, and the potential for screening therapeutics in the treatment of cognitive disorders in humans.

This Research Topic also highlights recent findings in our understanding of sleep and the clock to human cognitive function. Gaggioni et al. (2014) summarize work that provides evidence for an interactive relationship of sleep homeostasis and circadian rhythmicity on cognitive brain activity in humans. Lemos et al. (2014) show naps are able to enhance memory in school-aged adolescents, evidence supporting sleep in facilitating memory. Finally, Bellesi et al. (2014) review work supporting the role of slow-wave sleep in cognitive performance, and provide an overview of methodological tools aimed at enhancing slow-waves in humans.

This Research Topic underscores the importance of using multiple model systems to broaden our understanding of the relationship between sleep, clocks, and memory. Basic mechanistic findings, taken from studies across species, will have important clinical relevance to our ever increasing 24-h society.

## Conflict of interest statement

The authors declare that the research was conducted in the absence of any commercial or financial relationships that could be construed as a potential conflict of interest.
